# Modified palatal flap via soft palate for skull base reconstruction

**DOI:** 10.31744/einstein_journal/2025AO1247

**Published:** 2025-04-17

**Authors:** Rogerio Pezato, Aldo Cassol Stamm, Andrea Santos Dumont Costacurta, Carlos Henrique Amaro Bravo Baptista, Reginaldo Raimundo Fujita, Camila Soares Dassi, Richard Louis Voegels, Andrew Thamboo, Miguel Soares Tepedino

**Affiliations:** 1 Division of Otolaryngology Head and Neck Surgery University of British Columbia Vancouver Canada Division of Otolaryngology Head and Neck Surgery, University of British Columbia, Vancouver, Canada.; 2 ENT Research Lab Department of Otorhinolaryngology and Head and Neck Surgery Universidade Federal de São Paulo São Paulo SP Brazil ENT Research Lab, Department of Otorhinolaryngology and Head and Neck Surgery, Universidade Federal de São Paulo, São Paulo, SP, Brazil.; 3 Rhinology and Skull Base Division Universidade do Estado do Rio de Janeiro Rio de Janeiro RJ Brazil Rhinology and Skull Base Division, Universidade do Estado do Rio de Janeiro, Rio de Janeiro, RJ, Brazil.; 4 Department of Ophthalmology and Otorhinolaryngology Universidade de São Paulo São Paulo SP Brazil Department of Ophthalmology and Otorhinolaryngology, Universidade de São Paulo, São Paulo, SP, Brazil.

**Keywords:** Skull base, Flap, Palate, Soft, Cranial fossa, Posterior, Paranasal sinuses, Cadaver, Nasal surgical procedure, Tomography, X-ray computed

## Abstract

**Objective:**

Given the importance of vascularized flaps, this study presents a new technique for reconstructing skull defects using a pedicled palatal flap via the soft palate.

**Methods:**

Five preserved cadaveric specimens were used to demonstrate the sequential steps involved in harvesting the palatal flap. In addition, 20 normal paranasal sinus computed tomography scans were analyzed to determine potential measurements of the flap (area, perimeter, greatest anteroposterior distance, and greatest transverse distance).

**Results:**

The average flap area in females and males was 11.8cm^2^ and 12.7cm^2^, respectively. The average perimeter in females and males was 13.5cm and 14.0cm, respectively. In all cases, the flap reached the infratemporal fossa and lower clivus.

**Conclusion:**

The palatal flap via the soft palate described in this study proved to be a metrically viable alternative in cadaveric and tomographic studies for use in endonasal surgeries, as well as for skull base and adjacent regions.

## In Brief

This study presents a new technique for reconstructing the skull base defects (clivus and infratemporal fossa) using a pedicled palatal flap via the soft palate.

## Highlights

■ The use of a pedicled palatal flap via the soft palate offers the advantage of low harvesting complexity.
■ The flap demonstrates highly positive metric data supporting its technical feasibility for reconstructing the skull base and adjacent regions.
■ Drilling is not necessary to enlarge the greater palatine foramen in this technique, which reduces the chances of injury to the vascular pedicle and surgical time.

## Graphical abstract

**Figure:**
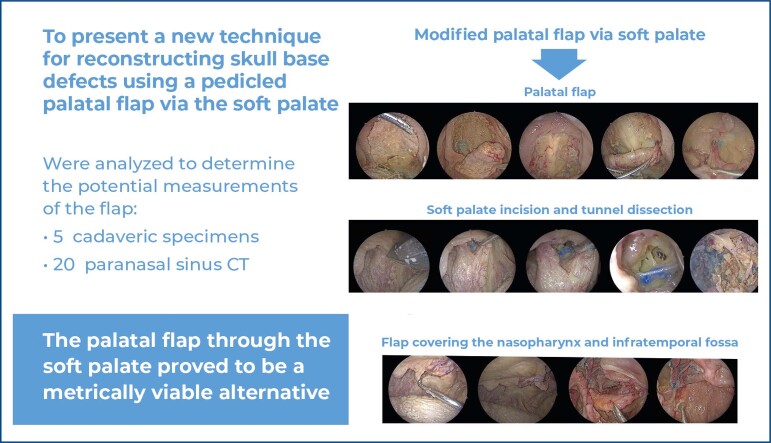

## INTRODUCTION

The surgical management of skull base pathologies presents considerable challenges. The external approach has been considered the gold standard for accessing this region for many decades despite its numerous complications and difficulties.^1,2^ In 1971, Donald Wilson introduced the concept of “keyhole surgery,” which emphasized limited surgical exposure of the brain. Building on this idea, approximately 20 years later, the expanded endonasal approach (EEA) began to gain acceptance as an optimal method for skull base surgery, offering direct access to the skull base without requiring brain retraction.^3^

Indications for the EEA have evolved anatomically beyond the sphenoid and sella. To evaluate the safety of EEA procedures, Kassam et al. published a series of 800 procedures performed from 1998 to 2007, in which surgical complications were analyzed.^4^ The transition to vascularized mucosal flaps for the reconstruction of skull base defects marked a milestone achievement, significantly reducing the rates of postoperative cerebrospinal fluid leaks.^5-7^ Furthermore, vascularized flaps play a secondary role in covering and protecting critical structures, such as the optic chiasm, optic nerves, and internal carotid arteries, which may be exposed following the tumor resection.

Considering its surface area and reliable vascular supply, the nasoseptal flap (NSF) is the cornerstone of the EEA reconstruction of the skull base.^7^ Despite its relevance, the NSF may not be available in cases of previous nasal surgery, tumor invasion, or damage to the pedicle during the approach.^5,8,9^To develop an alternative to the NSF, several types of vascularized flaps for endonasal skull base surgeries have been described in recent years, including the inferior turbinate flap, temporoparietal fascia flap, modified palatal flap, lateral wall, and lower sphenoid wall (upper tongue flap).^5,8-12^ It is important to highlight that parameters such as size and location of the defect, tissue similarity, and donor site morbidity are essential when selecting the type of flap to be used.^5,13-15^

Owing to the importance of vascularized flaps in skull base surgeries and the challenges involved in executing established techniques, expanding the number of available options is essential for tailoring the best reconstructive treatment for each case. This study presents a new technique for reconstructing skull base defects (clivus and infratemporal fossa) using a pedicled palatal flap via the soft palate.

## OBJECTIVE

This study aims to present a new technique for reconstructing skull base defects using a pedicled palatal flap via the soft palate.

## METHODS

Five preserved cadaveric specimens were used for anatomical dissection, which was originally performed at IRCAD # I01/25, Rio de Janeiro (RJ, Brazil). All specimens were prepared using a standardized protocol, which included vascular silicone injections colored red for arteries and blue for veins. The feasibility of harvesting and transposing a pedicled palatal flap through the soft palate was investigated. A navigation system (Fusion ENT Navigation, Medtronic^®^) was used to identify the greater palatine foramen, pterygoid foramen, foramen rotundum and foramen ovale.

Additionally, 20 normal paranasal sinus computed tomography (CT) scans were analyzed to determine potential flap measurements.

### Surgical technique

**Figure 1:**
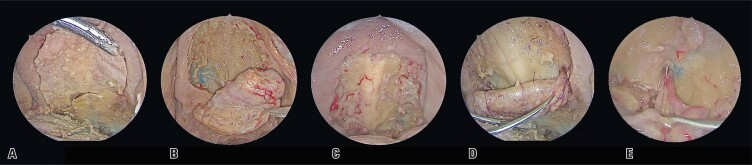
Demonstration of sequential steps for harvesting the palatal flap. A) Palatal incision; B and C) Donor area; D and E) Pedicle of the greater palatine artery

The palatal flap was created under endoscopic visualization, following the technique described by Ward.^15^ Using a 0° endoscope, a McIvor mouth opener was positioned, and incisions were made with a 15-blade scalpel along the entire gingival edge, approximately 2 mm from the dental elements (Figure 1A).

The mucosal flap was elevated by dissecting the mucosa from anterior to posterior in the subperiosteal plane of the hard palate, preserving one of the neurovascular pedicles emerging from the greater palatine foramen (Figure 1B to E).

**Figure 2:**
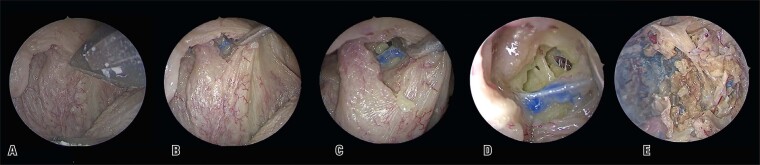
Demonstration of the tunnel creation for passing the flap. A) Incision in the soft palate; B, C and D) Tunnel dissection

An incision was made 3 mm superior to the glossopalatine arch using a 12-blade scalpel, ipsilateral to the preserved pedicle of the greater palatine artery (Figure 2A). After identifying the palatoglossus muscle with curved Kelly forceps, the anterior surface of the muscle was dissected superiorly to create a tunnel, extending until it reached the nasal cavity at the junction of the hard and soft palates (Figure 2B to D).

**Figure 3:**
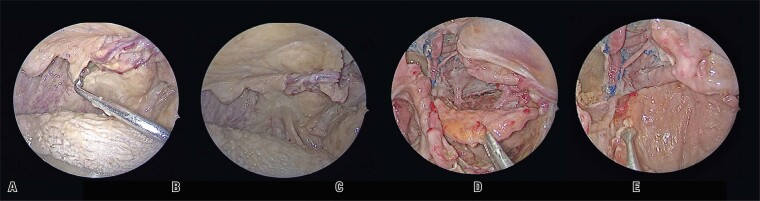
A and B) Flap repositioning through the tunnel; C and D) Flap covering the nasopharynx and infratemporal fossa

The flap was then transposed through the tunnel (Figure 3A and B). This palatal flap can be used in the EEA to cover the clival and infratemporal regions (Figure 3C and D). Care must be taken to avoid rotation of the flap along its axis during this stage and prevent compression of the vascular pedicle.

### Assessment of the size of the palatal flap

**Figure 4:**
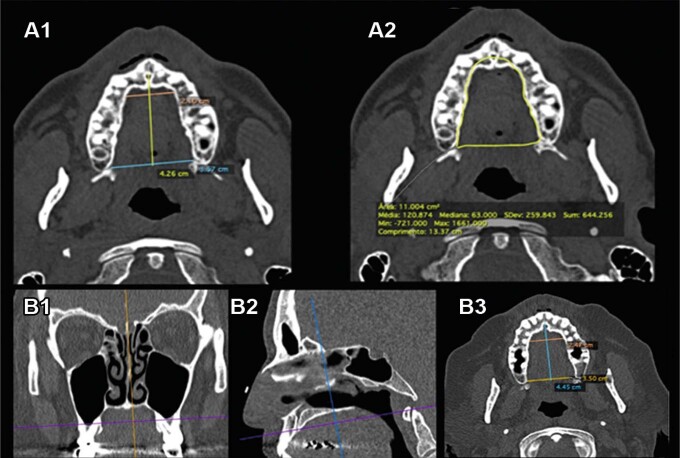
Demonstration of CT measurements of the palatal flap. A) Greatest anteroposterior distance, greatest transverse distance, area and perimeter; B) Alignment of cutting planes

Using 20 normal non-contrast paranasal sinus CT scans, the palatal flap area, perimeter, greatest anteroposterior distance, and greatest transverse distance were measured (Figure 4A and B). Sagittal and coronal CT reconstructions were generated from the axial images.

### Statistical analysis

Descriptive analysis of the flap dimensions was performed using mean, median, standard deviation, and maximum and minimum values. To compare differences between flap dimensions by sex, the Mann-Whitney U test was applied, with a p<0.05.

## RESULTS

### Creation and positioning of the palatal flap

The subperiosteal palatal mucosa was successfully detached without rupture in the five cadaver heads, and the greater palatine artery was identified bilaterally. In all the cases, the flap extended to the infratemporal fossa and lower clivus.

### Flap dimensions on CT

A total of 20 CT scans were analyzed, with half of the patients being male (10) and the other half women (10). The patients’ ages ranged from 18 to 70 years.

The measurements used to evaluate the flaps included area, perimeter, greatest anteroposterior, and greatest transverse distances. All data obtained are shown in table 1.

**Table 1:** CT measurements of the Palate flap(10 female, 10 male)

	Area (cm^2^)	Perimeter (cm)	Longer anteroposterior distance (cm)	Longer transverse distance (cm)
Mean				
Female	11.8	13.5	4.18	3.58
Male	12.7	14.0	4.24	3.63
Median				
Female	11.3	13.6	4.17	3.51
Male	12.1	13.7	4.26	3.57
Standard deviation				
Female	1.66	0.86	0.24	0.24
Male	1.52	0.87	0.22	0.28
Minimum				
Female	10.0	12.3	3.69	3.28
Male	11.0	13.0	3.93	3.29
Maximum				
Female	15.1	14.9	4.64	4.07
Male	15.0	15.4	4.68	4.13

There was a statistically significant difference in flap dimensions between the sexes. The average flap area in female patients differed by almost 1cm^2^ compared to that in male patients (11.8cm^2^ and 12.7cm^2^, respectively, p=0.03). The average perimeter also differed between the groups (13.5cm and 14.0cm, respectively, p=0.04).

The means of the greatest anteroposterior and transverse distances were very similar between the sexes, with variations of 0.06 cm and 0.05 cm, respectively.

## DISCUSSION

The use of various types of vascularized flaps in skull base surgery and EEA aims to protect critical structures and reduce the risk of serious complications. Their application represents a significant advancement in the management of postoperative morbidities, and the development of new flaps must be carefully evaluated.

The size and location of the defect, tissue similarity, and donor site morbidity are essential factors for choosing the appropriate flap,^5,13-15^ especially at the skull base, where dimensions are limited. In such cases, the use of palatal mucosa becomes an excellent option when nasal mucosa is unavailable.

The palatal flap described here is mainly nourished by the greater palatine artery, which is a branch of the maxillary artery. This technique is similar to that described by Oliver et al.;^11^ however, it was modified to transpose the flap through the soft palate rather than through the greater palatine foramen. As a result, drilling to enlarge the greater palatine foramen is not necessary, reducing the risk of injury to the vascular pedicle and surgical time.

Another advantage of the palatal flap is its low harvesting complexity. In addition to an otolaryngologist, general surgeons and even dentists will be able to assist in this phase of the surgery.

Regarding the area of the flap, a greater flap size in male patients was expected, considering the average anatomical difference between males and females. However, it is important to highlight that the maximum flap area observed in both groups was nearly the same, with a slight increase in females. This finding supports the idea that not all patients adhere to preestablished anatomical standards.

Despite causing greater morbidity, such as pain and dysphagia, in the postoperative period compared to a nasoseptal flap, the palatine flap remains an attractive option when other flaps with lower morbidity cannot be used or to complement the reconstruction of large defects.

This article presents an innovative flap alternative, supported by highly positive metric data, which demonstrates the technical feasibility of its use in skull base and adjacent region reconstruction. Further studies are required to confirm the feasibility of using the described flap *in vivo*.

## CONCLUSION

The palatal flap via the soft palate, as described here, has proven to be a viable alternative in cadaveric and tomographic studies for endonasal surgery, skull bases, and adjacent regions.
